# Experimental Confirmation of a Topological Isomer of the Ubiquitous Au_25_(SR)_18_ Cluster in the Gas Phase

**DOI:** 10.1021/jacs.0c11509

**Published:** 2021-01-14

**Authors:** Elina Kalenius, Sami Malola, María Francisca Matus, Rania Kazan, Thomas Bürgi, Hannu Häkkinen

**Affiliations:** † Department of Chemistry, Nanoscience Center, University of Jyväskylä, FI-40014 Jyväskylä, Finland; ‡ Department of Physics, Nanoscience Center, University of Jyväskylä, FI-40014 Jyväskylä, Finland; § Department of Physical Chemistry, University of Geneva, 30 Quai Ernest-Ansermet, 1211 Geneva 4, Switzerland

## Abstract

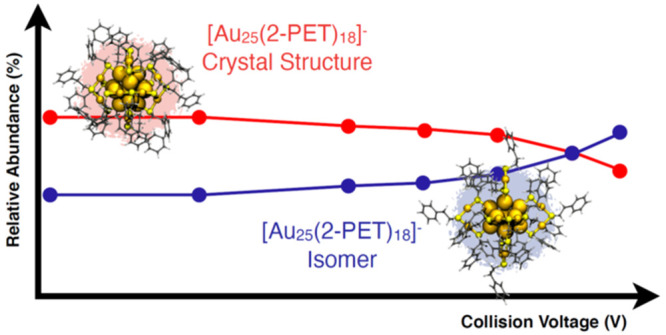

High-resolution electrospray ionization ion mobility mass spectrometry has revealed a gas-phase isomer of the ubiquitous, extremely well-studied Au_25_(SR)_18_ cluster both in anionic and cationic form. The relative abundance of the isomeric structures can be controlled by in-source activation. The measured collision cross section of the new isomer agrees extremely well with a recent theoretical prediction (MatusM. F.; et al. Chem. Commun.2020, 56, 808710.1039/d0cc03334k32543631) corresponding to a Au_25_(SR)_18_^–^ isomer that is energetically close and topologically connected to the known ground-state structure via a simple rotation of the gold core without breaking any Au–S bonds. The results imply that the structural dynamics leading to isomerization of thiolate-protected gold clusters may play an important role in their gas-phase reactions and that isomerization could be controlled by external stimuli.

Over the last two decades, research on monolayer-protected clusters (MPCs) has resulted in large progress.^1−3^ MPCs typically contain from a few atoms to a few hundred atoms in the metal core and are protected by a ligand shell. Their properties are strongly size-dependent and molecule-like because of the discrete electronic levels.^4^ MPCs are atomically precise. As such they are an attractive class of materials that allows studying the evolution of structure of matter as well as its properties as a function of size at the nanoscale.^5^ Furthermore, MPCs are considered for various applications, for example in sensing^6^ and catalysis,^7−9^ because of their tunable physical and chemical properties.

The tremendous progress made recently in the field is largely driven by the determination of structures by single-crystal X-ray diffraction (SCXRD). Since the first reported crystal structure of a thiolate-protected gold cluster, Au_102_(*p*MBA)_44_ (*p*MBA = *p*-mercaptobenzoic acid),^10^ numerous structures have been reported, mostly for gold and silver clusters^11^ but also for copper^12,13^ and alloy clusters^14^ protected by various ligands, including thiolates,^15^ phosphines,^16^ alkynyl groups,^17^ or N-heterocyclic carbenes.^18^ Given the continuing fast discovery of new structures, it seems that the community has so far just scratched the surface of a possibly vast structural space of stable MPCs.

An atomic structure determined by SCXRD provides a static snapshot of the cluster. However, there is evidence that MPCs are quite dynamic. For example, it has been shown that Au_38_(2-PET)_24_ (2-PET = phenylethylthiolate), a chiral cluster, can invert its handedness in solution.^19^ Simulations indicate that this is possible via concerted rotations of three gold atoms at the two poles of the metal core, which drag along the surface Au–SR–Au–SR–Au units.^20^ During this process no Au–S bonds are broken, in agreement with experiments, which reveal a low activation barrier. This example demonstrates that the potential energy surface that MPCs can explore is not restricted to one energy minimum (one structure). Given the flexibility of the Au–S framework of thiolate-protected gold clusters, it is somewhat surprising that there is hardly any evidence for isomers, i.e., clusters with the same composition but different structures of the Au–S framework. For thiolate-protected gold clusters, the only experimental demonstration of an isomer has been provided for Au_38_(2-PET)_24_. The two reported Au_38_ clusters^21,22^ have Au_23_ cores with different structures, and furthermore, they differ in the organization of the ligand shell. It should be noted that the two isomers of the cluster were prepared by separate synthetic routes. Recently, an interesting study reported interconversion between icosahedral- and face-centered cubic (fcc)-based metal cores in Au_144_(SC_2_H_2_Ph)_60_ imaged on a solid support by transmission electron microscopy (TEM). The conversion was concluded to be caused by electron irradiation during imaging.^23^

The reason for the lack of reported cluster isomers may be related to the difficulty of detecting them, especially if they are minor species. Isomers cannot be separated in ordinary mass spectrometry, and during the crystallization process only one isomer is selected. Isomerism is important since many measurements, e.g., the optical absorption spectrum, are ensemble measurements with contributions from all isomers. In extreme cases a minor isomer can even dominate certain properties. It is, for example, possible that a minor isomer shows much brighter fluorescence or a higher catalytic activity than the corresponding major isomer.

The possibility of coexisting cluster isomers that can interconvert should therefore be investigated. In fact, in a recent theoretical work^24^ some of us proposed an isomer of the Au_25_(SR)_18_ cluster anion (SR = thiolate) that is topologically connected to the isomer found in the crystal structure of the cluster.^25,26^ It should be noted that although Au_25_(SR)_18_ clusters are probably the most studied gold–thiolate cluster systems to date^27^ there has been no experimental indication of isomers. Simulations (ref (24)) indicated that isomerization of Au_25_(SR)_18_ takes place via a collective rotation of the icosahedral Au_13_ core, in a similar fashion as was described above for Au_38_(SR)_24_. According to the calculations, this new isomer has a significantly (ca. 20%) increased collision cross section (CCS), and it should therefore be detectable in the gas phase by ion mobility mass spectrometry (IM-MS).

Here we describe such experiments, in which we indeed found an isomer of Au_25_(2-PET)_18_ at significantly increased drift time with respect to the most abundant isomer. The increase in CCS is in excellent agreement with the theoretical prediction^24^ and additional simulations discussed below. We furthermore show that the relative abundance of the new (minor) isomer can be increased through collisional activation, which shows that isomerization can be controlled in the gas phase under our experimental conditions.

We reiterate here the prediction made in ref (24) and discuss new theoretical work studying the isomer structure and dynamics of Au_25_(2-PET)_18_ clusters in the gas phase. As was reported in ref (24), the gold–sulfur framework of the predicted topological isomer of Au_25_(2-PET)_18_^±^ is related to the corresponding crystal structure via a collective rotation of the icosahedral Au_13_ core in such a way that the six Au_2_(2-PET)_3_ units are bound to nearest-neighbor core Au atoms in the isomer (see Figure 1 and Video S1). Density functional theory (DFT) calculations found a low activation barrier of 0.6 eV for this transformation for Au_25_(SR)_18_^–^ when a simple methylthiolate was used as the model ligand.

**Figure 1:**
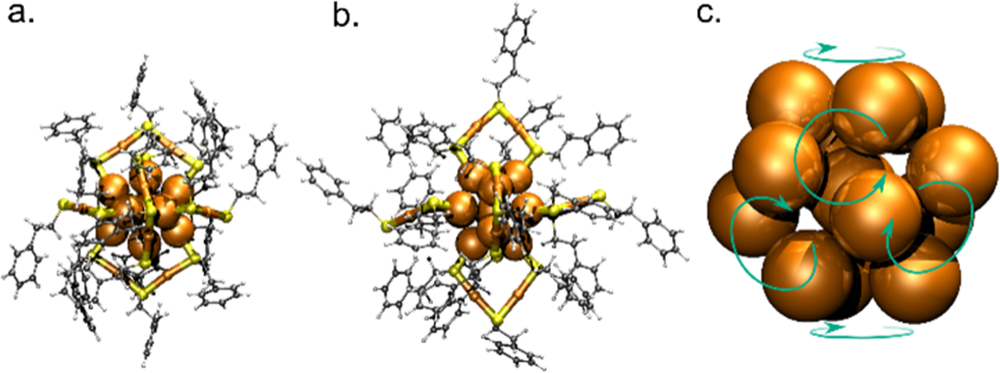
Structures of [Au_25_(2-PET)_18_]^−^. (a) Experimental crystal structure (main isomer).^25^ (b) DFT-relaxed (minor) isomer structure.^24^ (c) Illustration of the Au_13_ core transformation during the isomerization process.

Theoretical predictions of measurable CCS values and isomer energetics are affected by the level of DFT for electron–electron interactions and complications in dealing with the experimentally used ligand 2-PET (see details in the Supporting Information). The first predictions (ref (24)) were made by using the Perdew–Burke–Ernzerhof (PBE)^28^ functional, which is known to overestimate Au–Au bonds in the metal core by about 2–3%, yielding overestimations also for the geometrical cross sections and related CCS values. The simple local density approximation (LDA)^29^ reproduces experimental Au–Au distances well but does not yield reliable energetics. Additionally, ligand–ligand interactions in the 2-PET layer can contribute up to 1–2 eV in relative isomer energies depending on the detailed conformation of the ligands. Representative theoretical values for the geometrical cross sections are collected in Table S1. Irrespective of the DFT level, we find in Table S1 that the predicted geometrical cross sections for the isomers are consistently 16–22% larger than the values determined for structures based on experimental crystal data.

As discussed above, MPCs are dynamic systems. Currently, DFT computations are not practical for investigating fluctuations of the theoretical cross section values via molecular dynamics (MD) simulations. To this end, we resorted to MD simulations using the GROMACS 2019 software^30^ and our previously published classical force field for gold–thiolate clusters^31^ (see technical details in the Supporting Information). We performed extended 10 ns simulations in the gas phase at 300 K for three systems based on the corresponding crystal structures: [Au_25_(2-PET)_18_]^−^, [Au_25_(2-PET)_18_]^+^, and the cesium adduct [Au_25_(2-PET)_18_ + Cs]^+^. We note that the force field was parametrized^31^ using the crystal structure data and cannot reproduce the isomer transformation. However, the MD simulations gave here a unique first glimpse of the expected fluctuations of the ligand layer and their effects on the cluster’s “size”. We found that the three systems yield on average very similar radii of gyration (*R*_g_ = (7.5–7.6) ± (0.04–0.06) Å), correlating well with fluctuations in the theoretical geometrical cross sections. As an example, the time evolution of *R*_g_ and the geometrical cross section of the main isomer (average 468.1 ± 6.6 Å^2^) of Au_25_(2-PET)_18_^+^ are shown in Figure S7. Thus, fluctuations up to about 1.5% could be expected in the cross sections, including the Cs^+^ adduct, where the simulations showed interesting dynamics of the Cs^+^ cation with one to three phenyl rings of the ligands at any given time (Figure S8 and SI Video 2).

Samples of Au_25_(2-PET)_18_ (synthesized as negatively charged and neutral) were measured using electrospray ionization ion mobility mass spectrometry (ESI-IM-MS) using both polarizations. Experiments were performed using drift tube ion mobility mass spectrometry (DTIM-MS) with high-purity N_2_ as drift gas. The DTIM-MS method has certain benefits for MPC analysis compared with the more frequently used traveling wave ion mobility technique (TWIMS) and recently commercialized trapped ion mobility (TIMS).^32−36^ First, DTIM shows relatively high resolving power (*R* > 100 Ω/ΔΩ, meaning that ions with a difference in CCS of less than 2% can be separated), increasing the potential discovery of closely related isomeric structures.^37^ Second, in DTIM, the drift time of an ion is directly related to its CCS, and the ^DT^CCS_N_2__^38^ value can therefore be directly calculated from the measured drift time and other known experimental parameters without the need for calibration procedures.^39^

The sample synthesized as [Au_25_(2-PET)_18_]^−^ was measured on negative polarization and showed [Au_25_(2-PET)_18_]^−^ as a base peak at *m*/*z* 7393 (Figure 2a). The ion mobility arrival time distribution (ATD) for this ion shows the most intense drift peak at 58.3 ms, which we assign to the main isomer of [Au_25_(2-PET)_18_]^−^. This resulted in a ^DT^CCS_N_2__ value of 454.4 Å^2^, which corresponds well to the geometrical cross section (473 Å^2^) estimated earlier on the basis of the crystal structure of [Au_25_(2-PET)_18_]^−^.^24^ According to the extracted mass spectra, the smaller peaks (51.0, 53.0, and 43.6 ms) correspond to clusters (mainly doubly charged dimer and triply charged trimer) and their fragments (with fragmentation taking place in the drift tube). The formation of similar cluster species in the ESI source was observed previously by Pradeep et al.^34^ A small peak at 62 ms is observed only on negative polarization and results in a less than 40 Å^2^ (8% in relative size) increase corresponding to the main isomer. We assume this to originate from a stable unknown conformation of the ligand layer. The drift peak at 74.1 ms has not been observed in earlier studies and does not show any fragmentation in its extracted mass spectrum (see Figure S3). The experimental ^DT^CCS_N_2__ value for that ion is 581.7 Å^2^ (21.9% larger compared with the main structure). This value is in excellent agreement with the geometrical cross section of 583 Å^2^ estimated previously from DFT calculations for a low-energy isomer of [Au_25_(2-PET)_18_]^−^.^24^ We therefore assign the peak observed at 74.1 ms to this (minor) isomer of [Au_25_(2-PET)_18_]^−^ predicted by the calculations.

**Figure 2:**
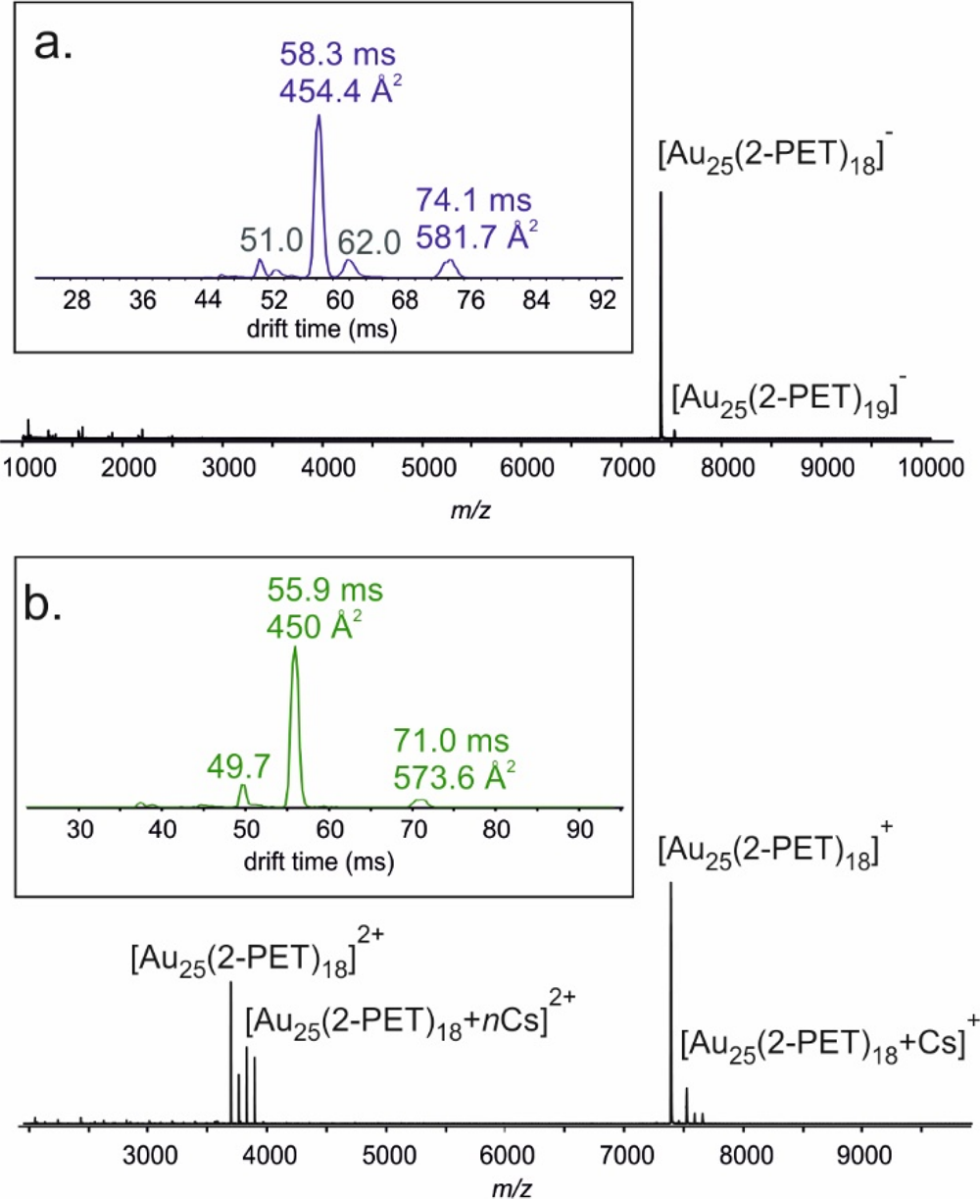
ESI-IM-MS spectra for samples of Au_25_(2-PET)_18_ in toluene/MeOH. The insets show arrival time distributions for most abundant peaks corresponding to [Au_25_(2-PET)_18_]^∓^. (a) Negative-mode mass spectrum. (b) Positive-mode mass spectrum (measured with addition of CsOAc).

Using the cluster structures of ref (24), theoretical CCS values were calculated using the project approximation (PA) method^41,42^ (Table S2), and structures for the main and minor isomers resulted in ^PA^CCS_N_2__ values of 477.7 and 592.7 Å^2^, respectively. Although the ^PA^CCS_N_2__ values are slightly higher than the values derived from experiment, both theory and experiment predict an CCS increase of around 19% for the minor isomer, in good agreement with the DFT predictions.^24^

The sample synthesized as neutral Au_25_(2-PET)_18_ was measured on positive polarization with an excess of CsOAc to enhance ionization. The mass spectrum showed both 1+ and 2+ cations for naked Au_25_(2-PET)_18_ and multiple Cs^+^ adducts (Figure 2b). The base peak, [Au_25_(2-PET)_18_]^+^ at *m*/*z* 7393, shows an ATD very similar to that of the negative ion [Au_25_(2-PET)_18_]^−^. The main and minor isomers are observed at drift times of 55.9 and 71.0 ms, respectively. These drift times result in ^DT^CCS_N_2__ values of 450.0 and 573.6 Å^2^, respectively, again showing a similar relative difference (27.5%) as for negative ions. The project approximation resulted in a theoretical CCS values (^PA^CCS_N_2__) of 461.9 Å^2^ for the main isomer and 591.1 Å^2^ for the minor isomer (Table S2), a relative increase of 28.0%. The ^DT^CCS_N_2__ value of 450.0 Å^2^ for the main isomer compares very well to the geometrical cross section of 458 Å^2^ for the crystal structure of the cationic cluster (ref (24)), and the value of 573.6 Å^2^ compares well to the predicted geometrical cross section of 552–586 Å (depending on the level of DFT) for the minor isomer in Table S1.

In-source activation for both samples was done by increasing the collision voltage at the fragmentor lens placed at the end of the ion transfer capillary.^43^ Similar experiments have been previously used to induce unfolding of proteins (CIU experiments),^44−47^ but this is the first such experiment to study MPCs and follow the dynamics between different isomers. Measurements on both polarities show the abundance of the main isomer to decrease upon activation, whereas the abundance of the minor isomer showed the opposite behavior (Figures 3 and S5), implying the transformation of the main isomer into the minor isomer. Our results thus strongly indicate that isomerization is possible in the gas phase under our experimental conditions. It is interesting to note that very recently it has been hypothesized that a similar topological isomer of an alkynyl-protected PtAu_24_ cluster can exist in the gas phase.^48^

**Figure 3:**
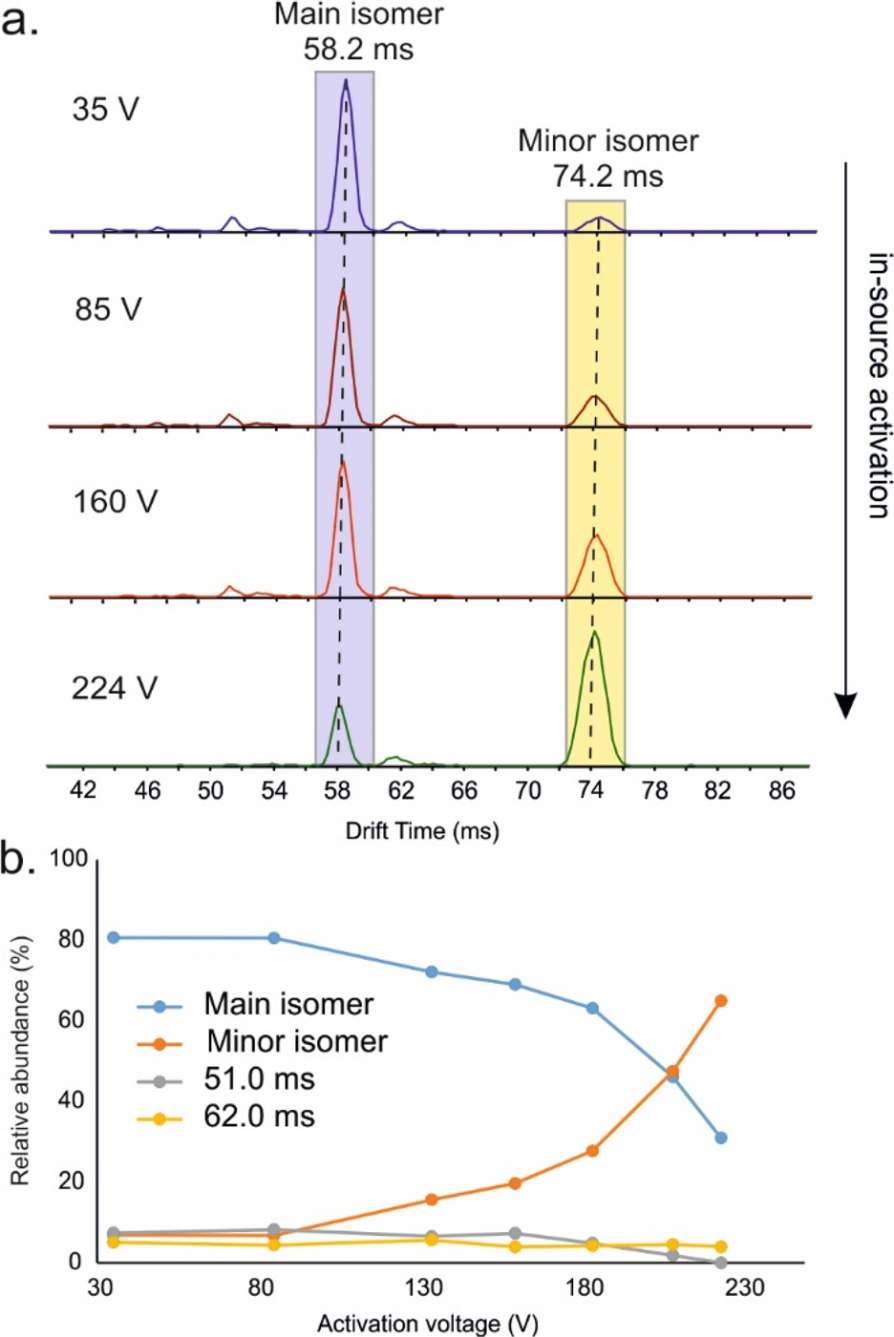
In-source activation of [Au_25_(2-PET)_18_]^−^. (a) IM arrival time distributions for the ion at *m*/*z* 7393 with different activation voltages. (b) Relative intensities of drift peaks as a function of activation voltage.

In conclusion, our experiments are the first to show that interconversion between isomers of thiolate-stabilized gold clusters can be controlled in the gas phase. This may open new avenues for detailed studies of the structural dynamics of MPCs that may be relevant to better understand their physical, optical, and chemical properties.

## Supporting Information Available

Detailed description of synthetic procedures and UV–vis spectra of [Au_25_(2-PET)_18_]^−^ and [Au_25_(2-PET)_18_]^0^ nanoclusters, technical details of the ion mobility mass spectrometry experiments, DFT calculations, and MD simulations, Figures S1–S8, and Tables S1 and S2 (PDF)
Animation showing the Au_13_ core transformation (MPG)
MD simulation of the Au_25_(2-PET)_18_ nanocluster with Cs^+^ adduct (MP4)

## Supplementary Material

ja0c11509_si_001.pdf

ja0c11509_si_002.mpg
