# Supplementary material for: Experimental Confirmation of a Topological Isomer of the Ubiquitous Au25(SR)18 Cluster in the Gas Phase
Source: J Am Chem Soc. 2021 Jan 14;143(3):1273–7. doi: 10.1021/jacs.0c11509 (PMC8023650; doi:10.1021/jacs.0c11509)
Supplement: Supplementary file 1 — ja0c11509_si_001.pdf [file ja0c11509_si_001.pdf]

Supporting Information for:

Experimental confirmation of a topological isomer of the ubiquitous $Au_{25}(SR)_{18}$ cluster in gas phase

Elina Kalenius,[†] Sami Malola,[‡] María Francisca Matus,[‡] Rania Kazan,[§] Thomas Bürgi,[§] Hannu Häkkinen[†,‡,*]

[†]*Department of Chemistry, Nanoscience Center, University of Jyväskylä, FI-40014 Jyväskylä, Finland.*

[‡]*Department of Physics, Nanoscience Center, University of Jyväskylä, FI-40014 Jyväskylä, Finland.*

[§]*Department of Physical Chemistry, University of Geneva, 30 Quai Ernest-Ansermet, 1211 Geneva 4, Switzerland.*

Table of contents

1. Experimental Section……………………………………………………………...S2
2. Computational Studies……………………………………………………………S5
3. Supplementary Figures…………………………………………………………....S6
4. Supplementary Tables…………………………………………………….…..…….S13
5. References……………………………………………………………….…………S14

1. Experimental Section

a) Reagents and Materials

All chemicals are commercially available and were used as received. Tetrachloroauric(III) acid trihydrate ($HAuCl_4 \cdot 3H_2O$, ≥ 49.0% (Au), Acros Organics), TOABr (Tetra-n-octylammonium bromide, 98%, Fluorochem) 2-phenylethanethiol (PhC_2H_4SH, ≥ 99%, Sigma-Aldrich), sodium borohydride ($NaBH_4$, 99%, Acros Organics). Tetrahydrofuran, acetone, methanol and toluene (analytical grade) were purchased from Fisher Scientific. The water used was ultrapure (resistivity 18.2 MΩ x cm).

b) Synthesis of $[Au_{25}(2\text{-PET})_{18}]^-$

$[Au_{25}(2\text{-PET})_{18}]^-$ was synthesized according to the method reported by Lu *et al.*[1] $HAuCl_4 \cdot 3H_2O$ (1 g, 2.54 mmol) and TOABr (982 mg, 3.05 mmol) were co-dissolved in 250 mL of THF and stirred vigorously over a period of ~30 min. The color of the solution changed from yellow to red. After that, PhC_2H_4SH (1.7 mL, 12.7 mmol) was slowly added to the reaction mixture at room temperature without changing the stirring speed. The reaction was allowed to stir for ~50 min, during which the color of the solution changed gradually from red to yellow and then to colorless. Next, a freshly prepared ice-cold solution of $NaBH_4$ (962 mg in 50.8 mL ultra-pure water, 25.4 mmol) was rapidly added to the flask, changing the solution color immediately to black, which indicated the formation of Au nanoclusters. The reaction was allowed to proceed for 32 hours at a constant stirring rate. It is worth mentioning that some white Au(I)-SR precipitates were also formed.

Afterwards, the reaction mixture was concentrated to a minimum volume on the rotovap, and a large amount of ice cold water was added to the flask. The solution was then gravity filtered, and the collected precipitate (Au_{25}^- nanoclusters) was thoroughly washed with methanol in order to remove the excess of thiols. After washing, acetone was used to dissolve and collect the Au_{25}^- nanocluster off the filter paper and the product was dried using a rotovap.

The product was then dissolved in a minimum amount of toluene before being passed over a Bio-Rad BioBeads SX-1 size exclusion chromatography (SEC) column in order to remove any residual

thiols, and any larger clusters that might be formed during the reaction, yielding highly pure $[Au_{25}(2\text{-PET})_{18}]^-$ nanocluster (Figure S1).

c) Synthesis of $[Au_{25}(2\text{-PET})_{18}]^0$

$[Au_{25}(2\text{-PET})_{18}]^0$ nanocluster was obtained according to a procedure reported by Antonello *et al.*[2] Briefly, the $[Au_{25}(2\text{-PET})_{18}]^-$ nanocluster was dissolved in DCM and passed over a silica gel column under aerobic conditions. The color of the cluster changed from dark orange to dark green indicating the conversion of the negatively charged cluster to its neutral form. The obtained cluster was dried and dissolved in a minimum amount of toluene before being passed over a SEC column in order to remove any impurities and residual charged species. A highly pure $[Au_{25}(2\text{-PET})_{18}]^0$ nanocluster is obtained as confirmed by UV-Vis spectroscopy (Figure S1).

d) Ion-Mobility Mass Spectrometry of $Au_{25}(2\text{-PET})_{18}$ Samples

Ion mobility mass spectrometry experiments were performed with Agilent 6560 ESI-IM-QTOF mass spectrometer equipped with dual AJS ion source, Drift Gas Upgrade Kit (Agilent Technologies, USA) and in-source activation fragmentor lens. High purity N_2 was used as drift gas directly from the gas cylinder (purity grade 6.0).

Solid samples of $Au_{25}(2\text{-PET})_{18}$ (anionic and neutral) were dissolved in toluene and diluted in toluene/MeOH 1:1. For positive polarization mass spectrometry measurements 5 % (v/v) of 50 mM CsOAc in MeOH was added to increase ionization efficiency.

The samples were injected into the ESI source with a flow rate of 5 µL min^{-1}. Dry gas temperature of 225 °C, drying gas flow rate 2 L min^{-1}, nebulizer pressure 20 psi, sheath gas temperature 225 °C, sheath gas flow 5 L min^{-1} were used. Capillary voltage of 5500 V, nozzle voltage of 2000 V and fragmentor voltage of 400 V were set as source parameters. The drift tube pressure was set to 3.95 Torr and high pressure funnel to 3.70 Torr. In the single-field IM experiments the drift tube entrance and exit voltages were set as 1700V and 224V, respectively. Trap filling time of 10 000 µs and trap release time of 350 µs were used. Collision cross-section (CCS) values were determined using multifield measurements and then drift tube entrance voltage was varied from

1650 V to 1950 V with 50 V increments. Higher entrance voltages (normally 1700 V) were necessary to ensure drift of all isomers within instrument maximum drift time of 133 ms. Before sample introduction, ES tuning mix (Agilent Technologies) was measured as a quality control sample for CCS values.[3,4] Data was analyzed using MassHunter Qualitative Navigator (B.09.00) and MassHunter IM-MS Browser (Version B.08.00) from Agilent Technologies, USA.

The commercially available Agilent 6560 instrument was modified by adding an additional fragmentor lens (also known as collision induced unfolding (CIU) lens) after transfer capillary to enable ion activation prior the drift tube.[5–7] The dc potential on this lens can be independently controlled to adjust the ion acceleration electric field. The activation voltage was defined as the voltage difference between the fragmentor lens and high pressure funnel entrance.

Theoretical CCS values were mainly calculated with IMoSSuite 1.10[8] and using experimental parameters (gas, temperature and pressure). Several theoretical approaches (project approximation (PA), trajectory method with Lennard-Jones parameters (TMLJ, TMLJQ), diatomic trajectory method (DTM) and exact hard sphere scattering (EHSS/DHSS)) were tested. Different Lennard-Jones parameters incorporated in different versions of IMoSSuite (1.06 and 1.10) were also compared. Best comparison to experimental data was obtained using project approximation, (Table S2), which implies that most of the more resource-demanding methods do not actually describe well interactions between Au clusters and N_2 drift gas. However, all methods predicted well relative increase of CCS between the main and minor isomers. This varied for cation between 25 and 34 % (experimental increase 27.5 %) and for anion between 19 and 23 % (experimental 21.9 %).

Coordinates for CCS calculations were obtained from the known published experimental crystal structures.[9,10] For the new topological cluster isomer we used the density functional theory (DFT)-relaxed model structure predicted by some of us for $[Au_{25}(2\text{-PET})_{18}]^q$ (q = -1, 0, +1).[11]

2. Computational Studies

a) Density Functional Theory (DFT) Calculations

The structures of main and minor isomers of $[Au_{25}(2\text{-PET})_{18}]^{+/-}$ clusters were relaxed by DFT. The code-package GPAW[12] was used with real-space grid and 0.2 Å grid spacing. Structures were relaxed using both PBE[13] and LDA[14] functionals. The relaxed DFT structures and a set of classical force field molecular dynamics (MD)-snapshot structures (see *section 2b)* below) were analysed for geometrical cross section in a similar way as described in Ref. 11. The initial structures for the clusters were taken either from crystal structures in case of main isomer or were originated from reactive-MD simulations in case of anion cluster of minor isomer.[11] The model structure for the minor isomer of the cation cluster was reconstructed by the ligand layer arrangement to match the original ligand binding directions found in the main isomer of the cationic cluster.

b) Gas Phase Molecular Dynamics (MD) Simulations

The PBE-relaxed structures of $[Au_{25}(2\text{-PET})_{18}]^-$, $[Au_{25}(2\text{-PET})_{18}]^+$, and a Cesium adduct $[Au_{25}(2\text{-PET})_{18} + Cs]^+$ (based on the corresponding crystal structures) were relaxed and dynamically simulated by using GROMACS 2019[15,16] MD simulation package with an AMBER force field for thiol−gold interactions.[17] For the initial configuration of the $[Au_{25}(2\text{-PET})_{18} + Cs]^+$ system, one Cs^+ cation was placed outside the cluster with a distance of 2 nm from the central Au atom.
Energy minimizations were carried out by using the steepest descent algorithm, followed by 10 ns of production MD. A leapfrog integrator with 2 fs time step, a velocity-rescale thermostat with a reference temperature of 300 K and a coupling time constant of 0.1 ps[18] were used. Periodic boundary conditions and pressure coupling were turned off. The center of mass motion (translational and rotational velocity around the center of mass) was removed to avoid the fast spin of the cluster. All bond lengths were constrained with the LINCS algorithm.[19] Cut-off electrostatics were applied by using as Coulomb cut-off value the same dimension of the cubic simulation box. Radius of gyration were computed for MD trajectories by using the *measure rgyr* command in VMD[20] with *weight* option. The masses were defined as 196.9665, 12.0107, 1.00794 and 32.0649 (amu) for Au, C, H and S, respectively.

3. Supplementary Figures

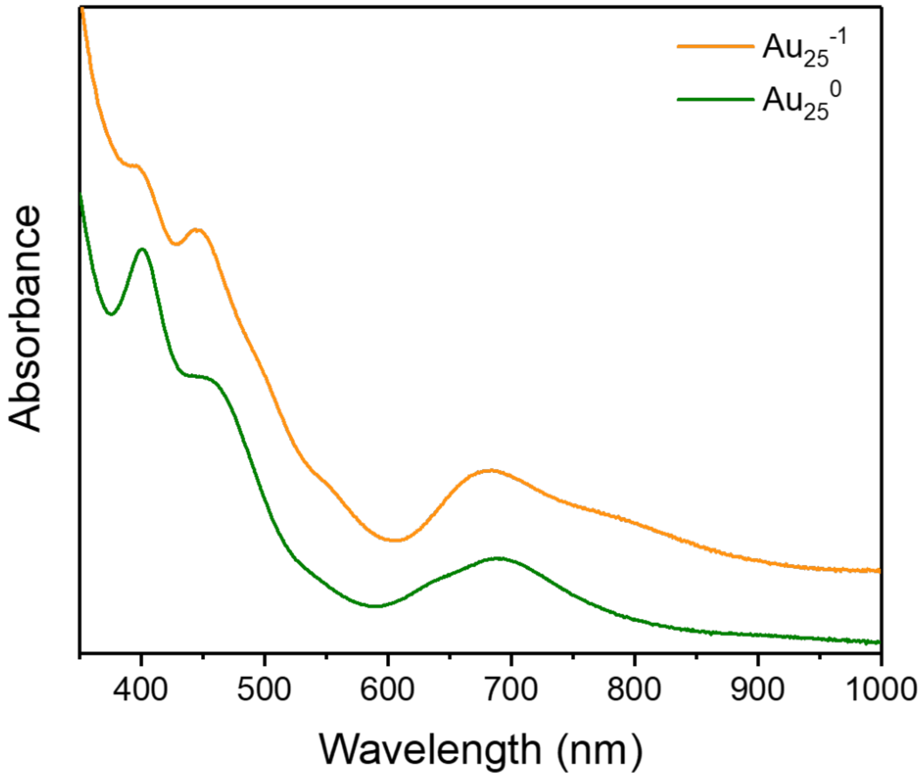

Figure S1. UV-Vis spectrum of pure $[Au_{25}(2\text{-PET})_{18}]^{-}$ and $Au_{25}(2\text{-PET})_{18}]^{0}$ nanoclusters in toluene. The spectra were normalized at 400 nm and off-set vertically for clarity.

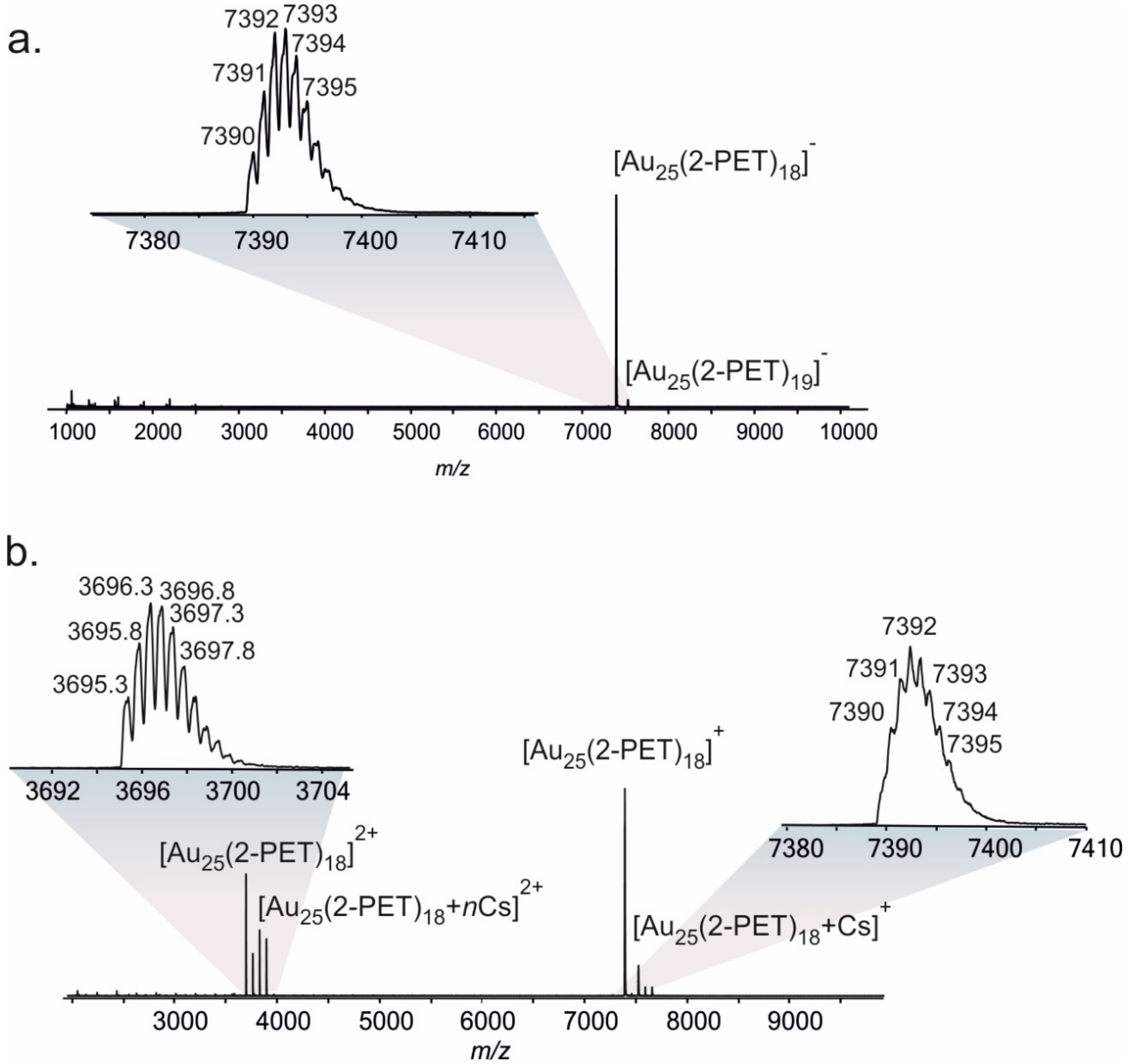

Figure S2. ESI-QTOF mass spectra of $Au_{25}(2\text{-}PET)_{18}$ nanocluster on a) negative polarization and b) positive polarization. Insets show zoomed views for main peaks.

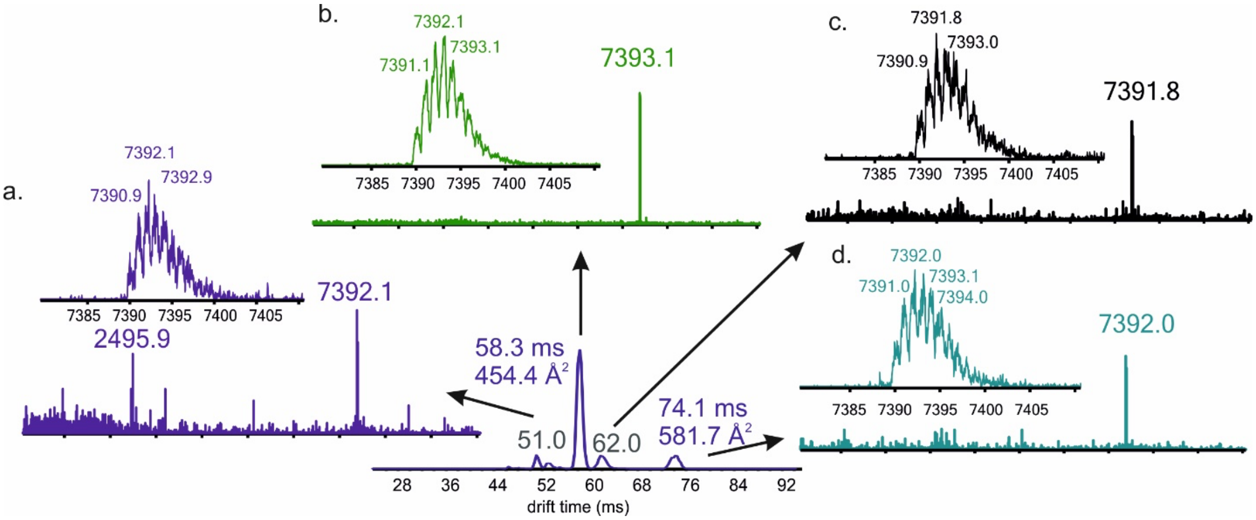

Figure S3. Extracted mass spectra from arrival time distribution of $[Au_{25}(2\text{-PET})_{18}]^-$ on negative polarization. Insets show extracted mass spectra from drift peaks a) 50.2-51.9 ms, b) 56.2-60.6 ms, c) 60.0-64.1 ms, and d) 71.9-76.2 ms.

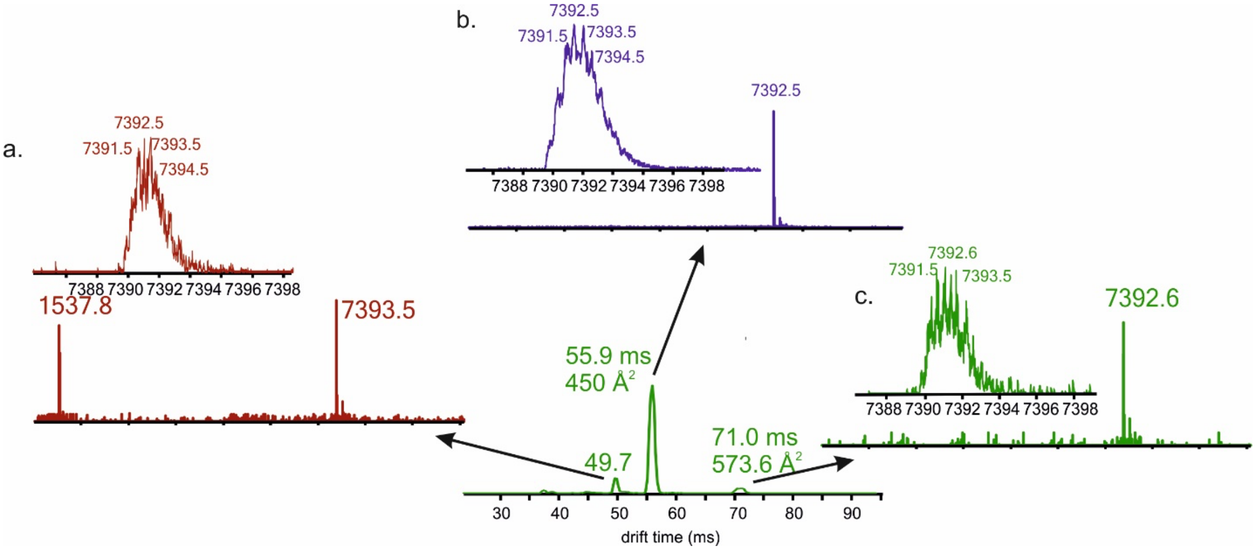

Figure S4. Extracted mass spectra from arrival time distribution of $[Au_{25}(2\text{-PET})_{18}]^+$ on positive polarization. Insets show extracted mass spectra from drift peaks a) 48.4-51.3 ms, b) 53.4-58.9 ms, and c) 68.7-72.8 ms.

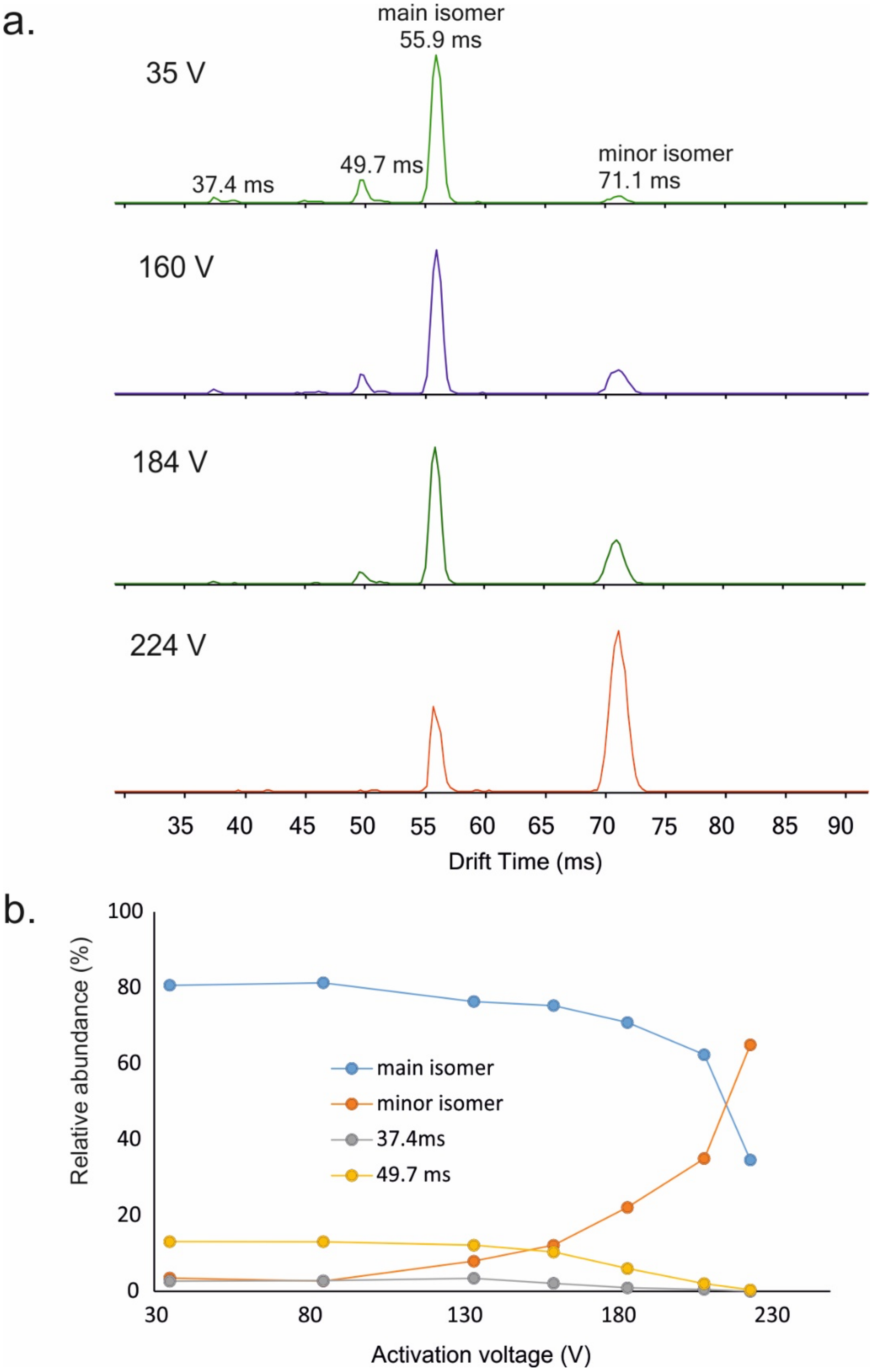

Figure S5. In-source activation of $[Au_{25}(2\text{-PET})_{18}]^+$ nanocluster. a) IM arrival time distributions for ion at *m/z* 7393 with different activation voltages. b) Relative intensities of drift peaks as a function of activation voltage.

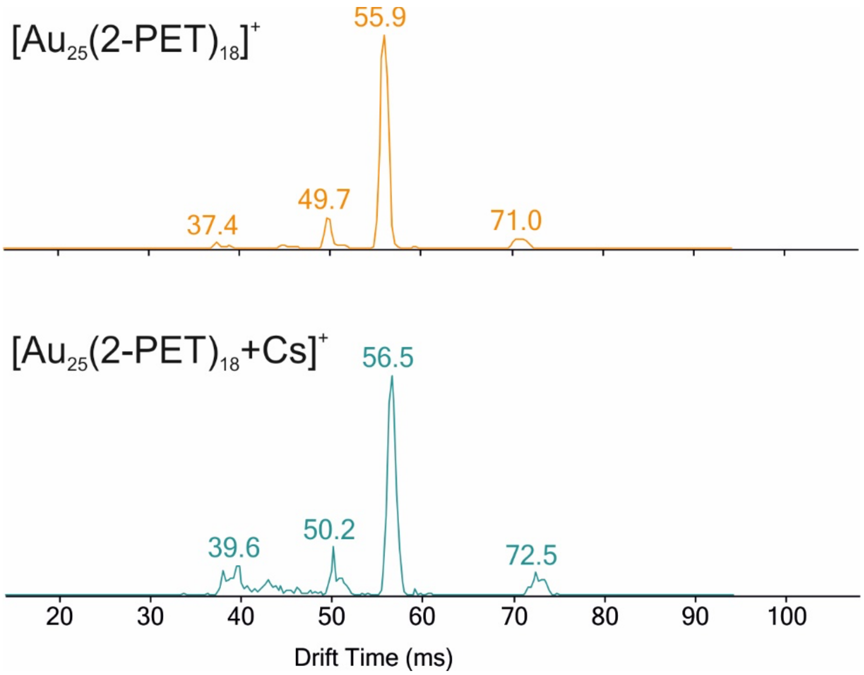

Figure S6. IM arrival time distributions for ions $[Au_{25}(2\text{-PET})_{18}]^+$ and $[Au_{25}(2\text{-PET})_{18}+Cs]^+$.

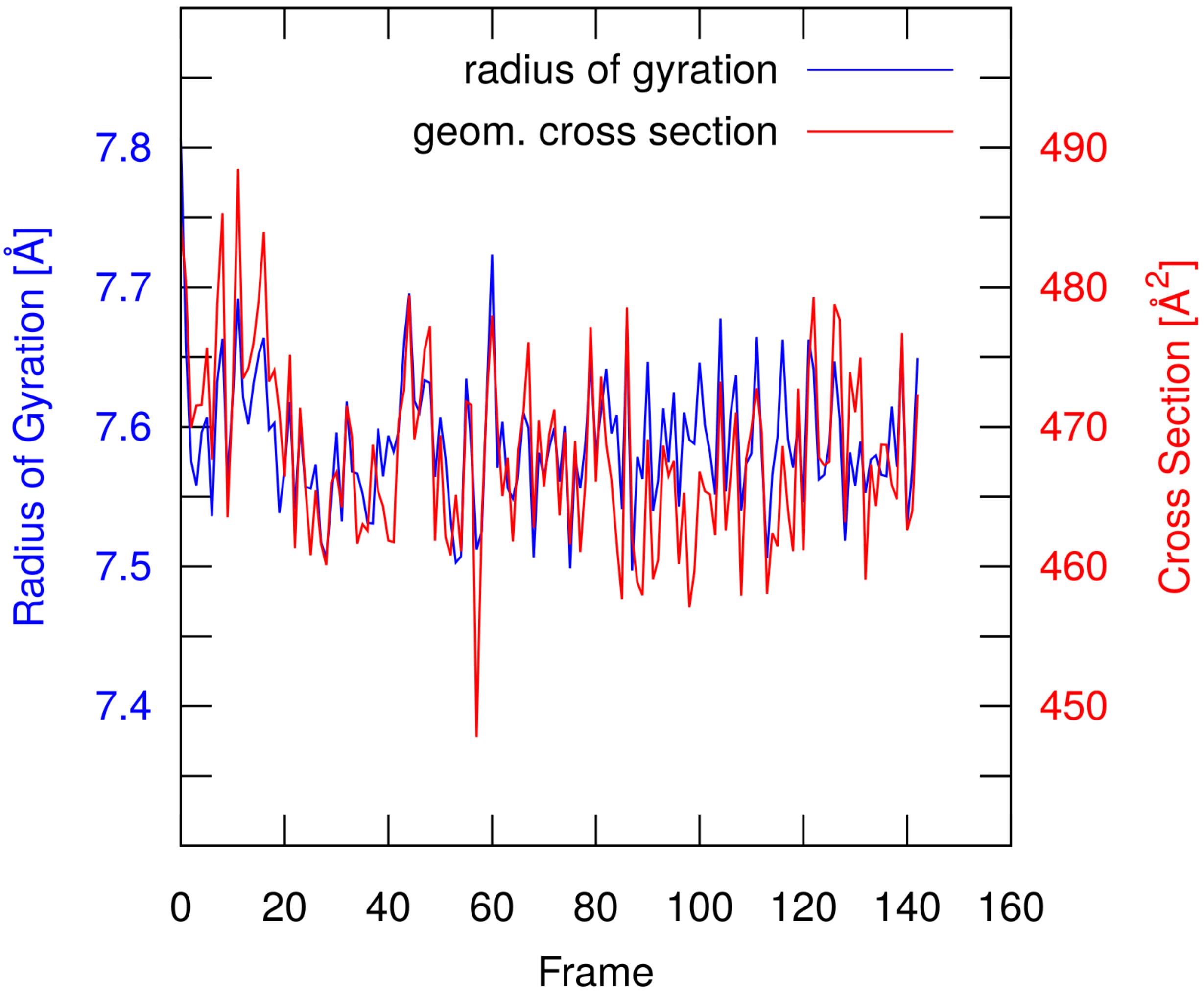

Figure S7. Fluctuations in the radius of gyration and geometrical cross section recorded in a GROMACS MD simulation of the main isomer of $[Au_{25}(2\text{-PET})_{18}]^+$ nanocluster in gas phase at 300 K. The analysed data consists of 143 configurations (frames) spanning 10 ns. The average values and standard deviations are 7.59 ± 0.05 Å and 468.1 ± 6.6 Å^2 for the radius of gyration and for the geometrical cross section, respectively. The fluctuations of these quantities are highly correlated.

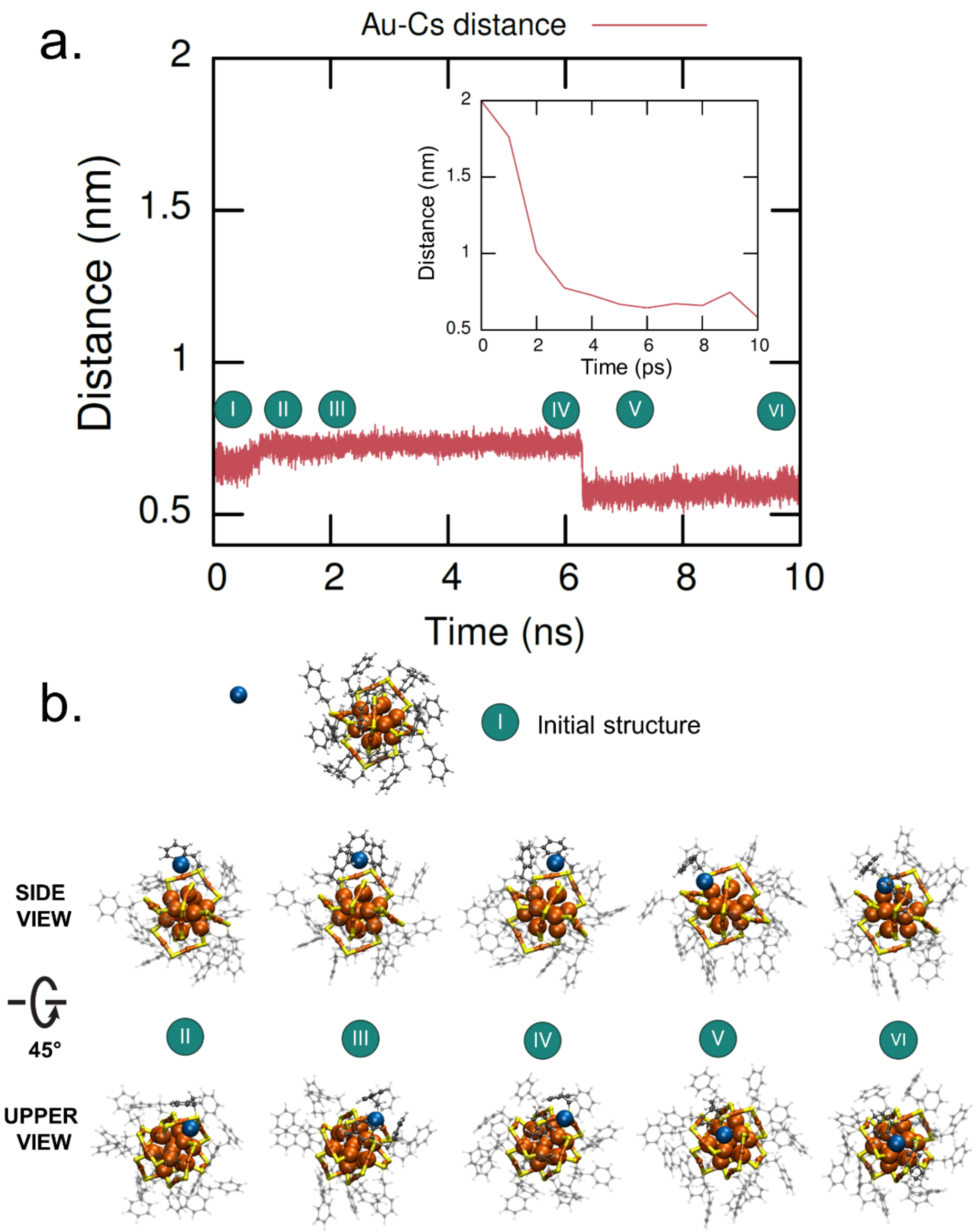

Figure S8. Analysis and visualization of a 10 ns GROMACS MD simulation of $[Au_{25}(2\text{-}PET)_{18}{+}Cs]^{+}$ nanocluster (main isomer) in the gas phase at 300 K. The top panel a) shows the time evolution of the Au-Cs^{+} distance. The bottom panel b) shows the initial structure (I) and a set of snapshots (II) - (VI) depicting the interaction of the Cs^{+} with 1-3 phenyl rings of the 2-PET ligands at various times. Au_{13} core and Cs^{+} are depicted as spheres, and the six RS-Au-SR-Au-SR units and 2-PET ligands with balls and sticks (Au: orange; S: yellow; C: gray; H: white; Cs: blue).

4. Supplementary Tables

Table S1. Theoretical geometrical cross section values for $Au_{25}(2\text{-}PET)_{18}$ nanoclusters using Density Functional Theory (DFT) calculations.

Nanocluster	Geometrical Cross Section (Å²)		
	Crystal Structure	PBE-relaxed Structure	LDA-relaxed Structure
$[Au_{25}(2\text{-}PET)_{18}]^-$ main isomer	473	504	472
$[Au_{25}(2\text{-}PET)_{18}]^-$ minor isomer	-	583	560
$[Au_{25}(2\text{-}PET)_{18}]^+$ main isomer	458	485	456
$[Au_{25}(2\text{-}PET)_{18}]^+$ minor isomer	-	586	552

Table S2. Experimental collision cross-section (CCS) values for $Au_{25}(2\text{-}PET)_{18}$ nanoclusters. The CCS values are calculated using DFT-relaxed models based on corresponding crystal structures (Table S1).

Nanocluster	Exp. $^{DT}CCS_{N2}$	Theoretical CCS_{N2} (Å²)					
		PA	EHSS/DHSS	TMLJQ	TMLJ (1.10)*	TMLJ (1.06)*	DTM
$[Au_{25}(2\text{-}PET)_{18}]^-$ main isomer	454.4	477.7	551.1	630.6	641.9	615.2	525.7
$[Au_{25}(2\text{-}PET)_{18}]^-$ minor isomer	581.7	592.7	716.2	786.0	795.5	781.1	686.4
$[Au_{25}(2\text{-}PET)_{18}]^+$ main isomer	450.0	461.8	532.5	627.7	632.5	508.2	508.2
$[Au_{25}(2\text{-}PET)_{18}]^+$ minor isomer	573.6	591.1	714.7	785.3	796.1	777.1	684.0

*1.10 and 1.60 refer to different Lennard-Jones parameters incorporated in IMoSSuite version 1.10 and 1.06, respectively.

5. References

(1) Lu, Y.; Jiang, Y.; Gao, X.; Chen, W. Charge State-Dependent Catalytic Activity of [Au 25 (SC 12 H 25) 18] Nanoclusters for the Two-Electron Reduction of Dioxygen to Hydrogen Peroxide. *Chem. Commun.* **2014**, *50* (62), 8464–8467.

(2) Antonello, S.; Arrigoni, G.; Dainese, T.; De Nardi, M.; Parisio, G.; Perotti, L.; Rene, A.; Venzo, A.; Maran, F. Electron Transfer through 3D Monolayers on Au25 Clusters. *ACS Nano* **2014**, *8* (3), 2788–2795.

(3) Gabelica, V.; Shvartsburg, A. A.; Afonso, C.; Barran, P.; Benesch, J. L. P.; Bleiholder, C.; Bowers, M. T.; Bilbao, A.; Bush, M. F.; Campbell, J. L.; Campuzano, I. D. G.; Causon, T.; Clowers, B. H.; Creaser, C. S.; De Pauw, E.; Far, J.; Fernandez-Lima, F.; Fjeldsted, J. C.; Giles, K.; Groessl, M.; Hogan, C. J.; Hann, S.; Kim, H. I.; Kurulugama, R. T.; May, J. C.; McLean, J. A.; Pagel, K.; Richardson, K.; Ridgeway, M. E.; Rosu, F.; Sobott, F.; Thalassinos, K.; Valentine, S. J.; Wyttenbach, T. Recommendations for Reporting Ion Mobility Mass Spectrometry Measurements. *Mass Spectrom. Rev.* **2019**, *38* (3), 291–320.

(4) Stow, S. M.; Causon, T. J.; Zheng, X.; Kurulugama, R. T.; Mairinger, T.; May, J. C.; Rennie, E. E.; Baker, E. S.; Smith, R. D.; McLean, J. A. An Interlaboratory Evaluation of Drift Tube Ion Mobility–mass Spectrometry Collision Cross Section Measurements. *Anal. Chem.* **2017**, *89* (17), 9048–9055.

(5) Dixit, S. M.; Polasky, D. A.; Ruotolo, B. T. Collision Induced Unfolding of Isolated Proteins in the Gas Phase: Past, Present, and Future. *Curr. Opin. Chem. Biol.* **2018**, *42*, 93–100.

(6) Haler, J. R. N.; Massonnet, P.; Far, J.; de la Rosa, V. R.; Lecomte, P.; Hoogenboom, R.; Jérôme, C.; De Pauw, E. Gas-Phase Dynamics of Collision Induced Unfolding, Collision Induced Dissociation, and Electron Transfer Dissociation-Activated Polymer Ions. *J. Am. Soc. Mass Spectrom.* **2018**, *30* (4), 563–572.

(7) Vallejo, D. D.; Polasky, D. A.; Kurulugama, R. T.; Eschweiler, J. D.; Fjeldsted, J. C.; Ruotolo, B. T. A Modified Drift Tube Ion Mobility-Mass Spectrometer for Charge-Multiplexed Collision-Induced Unfolding. *Anal. Chem.* **2019**, *91* (13), 8137–8146.

(8) Larriba-Andaluz, C.; Hogan Jr, C. J. Collision Cross Section Calculations for Polyatomic Ions Considering Rotating Diatomic/linear Gas Molecules. *J. Chem. Phys.* **2014**, *141* (19),

194107.

(9) Heaven, M. W.; Dass, A.; White, P. S.; Holt, K. M.; Murray, R. W. Crystal Structure of the Gold Nanoparticle [N (C8H17) 4][Au25 (SCH2CH2Ph) 18]. *J. Am. Chem. Soc.* **2008**, *130* (12), 3754–3755.

(10) Tofanelli, M. A.; Salorinne, K.; Ni, T. W.; Malola, S.; Newell, B.; Phillips, B.; Häkkinen, H.; Ackerson, C. J. Jahn–Teller Effects in Au 25 (SR) 18. *Chem. Sci.* **2016**, *7* (3), 1882–1890.

(11) Matus, M. F.; Malola, S.; Bonilla, E. K.; Barngrover, B.; Aikens, C. M.; Häkkinen, H. A Topological Isomer of the Au25 (SR) 18-Nanocluster. *Chem. Commun.* **2020**, *56*, 8087–8090.

(12) Enkovaara, J. e; Rostgaard, C.; Mortensen, J. J.; Chen, J.; Dułak, M.; Ferrighi, L.; Gavnholt, J.; Glinsvad, C.; Haikola, V.; Hansen, H. A. et al., Electronic Structure Calculations with GPAW: A Real-Space Implementation of the Projector Augmented-Wave Method. *J. Phys. Condens. Matter* **2010**, *22* (25), 253202.

(13) Perdew, J. P.; Burke, K.; Ernzerhof, M. Generalized Gradient Approximation Made Simple. *Phys. Rev. Lett.* **1996**, *77* (18), 3865.

(14) Perdew, J. P.; Wang, Y. Accurate and Simple Analytic Representation of the Electron-Gas Correlation Energy. *Phys. Rev. B* **1992**, *45* (23), 13244.

(15) M.J. Abraham, D. van der Spoel, E. Lindahl, B. Hess, and the G. development team. GROMACS 2019 http://www.gromacs.org.

(16) Van Der Spoel, D.; Lindahl, E.; Hess, B.; Groenhof, G.; Mark, A. E.; Berendsen, H. J. C. GROMACS: Fast, Flexible, and Free. *J. Comput. Chem.* **2005**, *26* (16), 1701–1718.

(17) Pohjolainen, E.; Chen, X.; Malola, S.; Groenhof, G.; Häkkinen, H. A Unified AMBER-Compatible Molecular Mechanics Force Field for Thiolate-Protected Gold Nanoclusters. *J. Chem. Theory Comput.* **2016**, *12* (3), 1342–1350.

(18) Bussi, G.; Parrinello, M. Stochastic Thermostats: Comparison of Local and Global Schemes. *Comput. Phys. Commun.* **2008**, *179* (1–3), 26–29.

(19) Hess, B.; Bekker, H.; Berendsen, H. J. C.; Fraaije, J. G. E. M. LINCS: A Linear Constraint Solver for Molecular Simulations. *J. Comput. Chem.* **1997**, *18* (12), 1463–1472.

(20) Humphrey, W.; Dalke, A.; Schulten, K. VMD: Visual Molecular Dynamics. *J. Mol. Graph. Model.* **1996**, *14* (1), 33–38.
